# Infantile Myofibroma Eroding into the Frontal Bone: A Case Report and Review of Its Histopathologic Differential Diagnosis

**DOI:** 10.1155/2012/630804

**Published:** 2012-08-27

**Authors:** Aatish Thennavan, Venkadasalapathi Narayanaswamy, Thanvir Mohammed Niazi, Lakshmi Rao, Raghu Radhakrishnan

**Affiliations:** ^1^Department of Oral and Maxillofacial Pathology, Manipal College of Dental Sciences, Manipal University, Manipal 576104, India; ^2^Orbit and Oculoplasty Clinic, Aravind Eye Hospital, Madurai 625020, India; ^3^Department of Oral Surgery, CSI College of Dental Sciences and Research, Madurai 625001, India; ^4^Department of Pathology, Kasturba Medical College, Manipal University, Manipal 576104, India

## Abstract

Infantile myofibroma is a rare and benign tumour of children presenting in the head and neck region. Rendering a final diagnosis of infantile myofibroma can be challenging in the light of nonspecific clinical, radiological findings and its histopathological similarities with a number of neoplasms especially spindle cell tumours. In this paper we discuss a case of infantile myofibroma in a 2-month-old infant, enumerating the various differential entities that have to be eliminated in reaching its specific diagnosis and highlighting the importance of immunopositivity to vimentin and smooth muscle actin (SMA) in establishing its myofibroblastic differentiation.

## 1. Introduction

Infantile myofibroma (IM) is a rare benign tumour of the myofibroblasts commonly found in the head and neck region of an infant. It usually manifests as a swelling in the dermis and subcutis with equal propensity of occurrence in male and female infants [1]. The swellings are frequently rubbery or firm, scar-like consistency with a size averaging from 0.5 to 1.5 cm. They are usually symptom-free with the internal lesions causing respiratory distress, vomiting, or diarrhoea, sometimes proving fatal. Radiographically, it appears as a well-defined unilocular radiolucency. Magnetic resonance imaging (MRI) is useful in planning the extent of surgery [2]. Microscopically, there is typically a biphasic pattern of light and dark staining areas owing to the difference in cellular morphology and arrangement. Depending on the predominance of cellular type, there can be a variety of lesions that come into its spectrum of differential diagnosis, namely, nodular fascitis, fibrous histiocytoma, neurofibroma, leiomyoma, and infantile fibromatosis causing confusion in reaching the final diagnosis in many instances [2]. The lesion is typically benign but because of difficulty in diagnosis may lead to inappropriate therapy.

We report a case of infantile myofibroma (IM) discussing its clinical, radiological, histopathological, and immunohistochemical features and the problems faced in its diagnosis.

## 2. Case report

**Figure 1:**
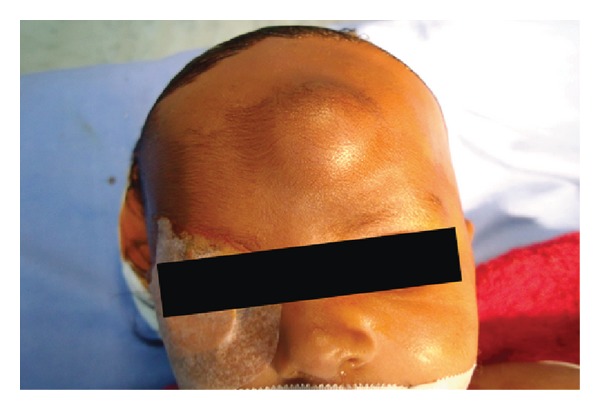
Swelling present on the forehead measuring 2.5 × 3 cm in dimension.

**Figure 2:**
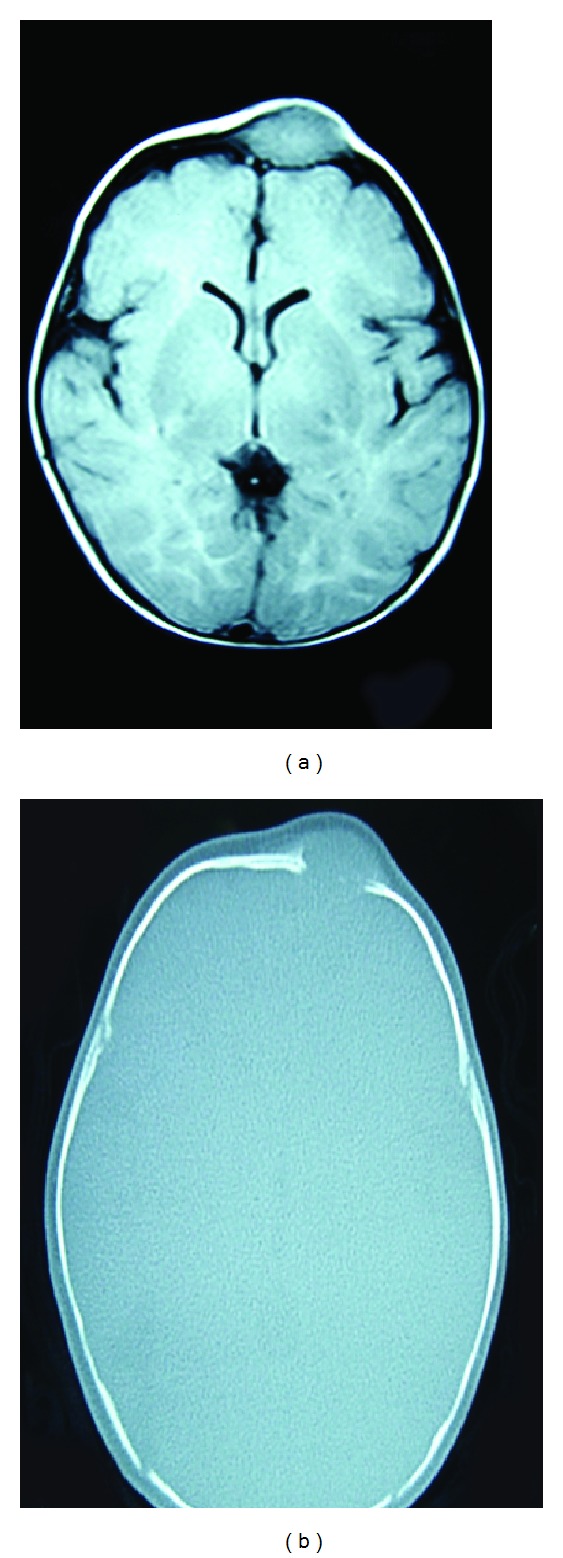
(a) T_2_ weighted image showing a well-defined mass, mildly hyperintense in comparison to brain and (b) T_1_ weighted image showing well-defined lesion with lytic cortical borders with homogenous enhancement.

A 70-day-old baby was noticed to have a persistent swelling on its forehead since birth by her mother. The pregnancy was normal with no history of trauma during delivery reported. The family history was also irrelevant. On clinical examination, the infant appeared systemically healthy with a firm, subcutaneous nodule situated in the right frontal bone region of the skull measuring 2.5 × 3 cm in dimension (Figure 1). The swelling was nontender with the overlying skin being noninflamed and normal in colour. MRI revealed an expansile soft tissue mass, oval in shape, and 2 × 3 cm in size with well-demarcated cortical borders mildly hyperintense in comparison with the brain matter (Figures 2(a) and 2(b)).

**Figure 3:**
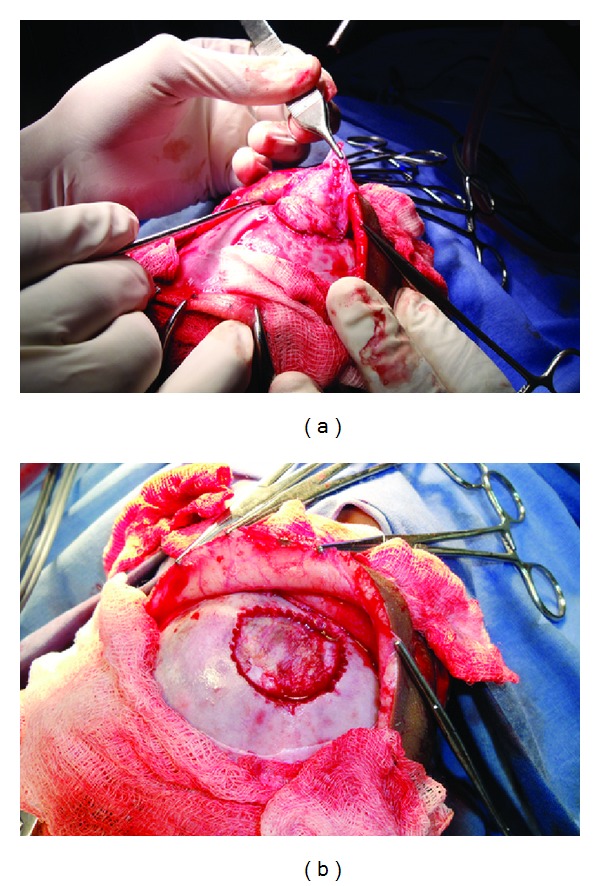
(a) Intraoperative image showing surgical excision of the lesion and (b) surgical site following excision.

**Figure 4:**
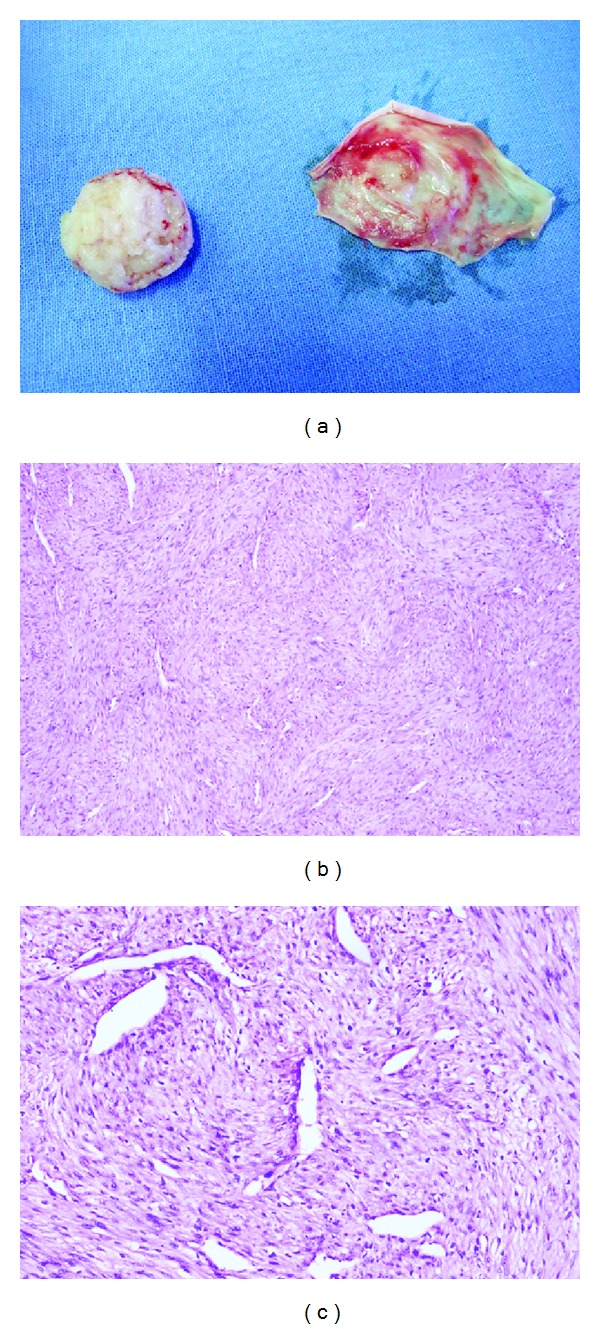
Excised specimen showing the periosteal lining (a), Photomicrograph showing tumour cells arranged in a biphasic pattern (H/E, ×40) (b), Photomicrograph showing light staining areas composed of plump myoid spindle cells arranged in short fascicles with elongated/cigar-shaped nuclei and dark staining areas composed of round or polygonal cells with slightly pleomorphic hyperchromic nuclei (H/E, ×100) (c).

Under ET, general anesthesia, supine position, a left supraorbital curvilinear incision was given to expose the lesion. Pericranium was elevated. The lesion was whitish, fleshy, and infiltrating the pericranium. Piecemeal total excision was done. Bony edges were nibbled. Dural surface was scrapped. Involved pericranium was excised and the wound was closed in layers (Figures 3(a) and 3(b)). Macroscopically, the mass appeared as a grayish-white gelatinous mass measuring 2 × 3 cm in dimension and was sent along with a piece of the periosteum covering the bone defect (Figure 4(a)) for histopathologic examination.

**Figure 5:**
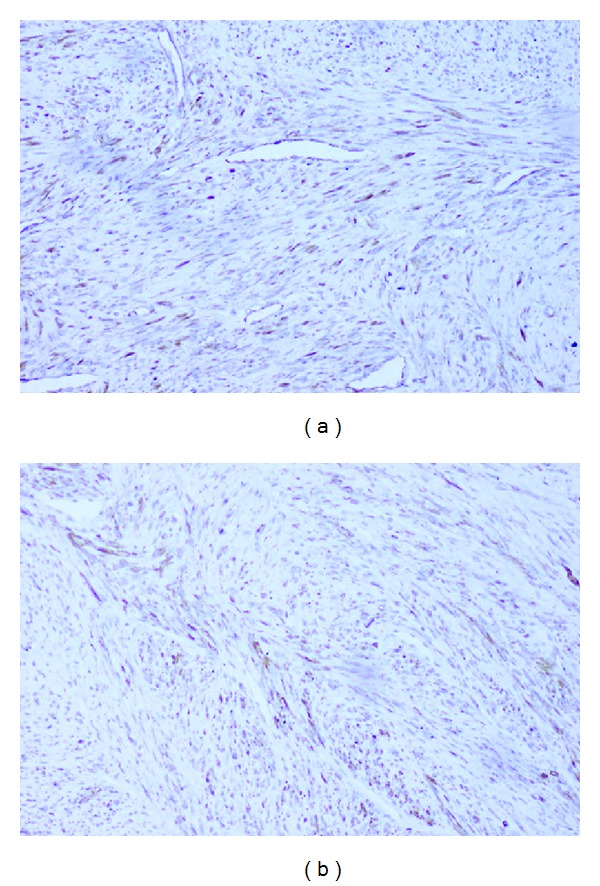
(a) Photomicrograph showing immunopositivity for vimentin (×100) and (b) photomicrograph showing immunopositivity for SMA (×100).

Microscopically, there was a multinodular growth pattern that had a biphasic pattern owing to the alternation of light and dark staining areas (Figure 4(b)). The light staining areas consisted mainly of spindle cells with eosinophilic cytoplasm arranged in short fascicles or whorls with elongated nuclei. The dark staining areas were composed of round cells with slightly pleomorphic hyperchromic nuclei around a distinct hemangiopericytoma-like vascular pattern (Figure 4(c)). Immunopositivity was seen for vimentin, SMA (Figures 5(a) and 5(b)), and negative for desmin, S-100 indicating a myofibroblastic lineage. Based on these characteristic features favouring a myofibroblast lineage, the lesion was diagnosed as infantile myofibroma.

## 3. Discussion

Myofibromatosis was initially described in 1951 as congenital fibrosarcoma [3] and subsequently as congenital generalized fibromatosis [4]. In 1965, congenital fibromatosis was classified into 2 types: a multiple form involving lesions of skin, subcutaneous tissue, skeletal muscle, and bone having a good prognosis and a generalized form involving visceral lesions and a poor prognosis [5]. Eventually, the role of myofibroblasts in its pathogenesis was revealed and these lesions were termed as “infantile myofibromatosis” [1]. The term “myofibroma” was agreed to be used when only one such lesion was present. Over the years, pathologists have preferred to use the terms “myofibroma” and “myofibromatosis” when describing these lesions with the prefix of “infantile” or “adult” indicating the age of presentation [2, 6]. Usually myofibromatosis is seen between birth and 2 years of age [7, 8]. Infantile hemangiopericytoma is now recognized as part of the spectrum of IM and may represent different stages of maturation of the same entity. 

The aetiopathogenesis of myofibroma is obscure. Some have reported its inheritance as an autosomal dominant pattern while others have suggested an autosomal recessive pattern [9, 10]. Intrauterine oestrogen hormone has been implicated in its genesis. Experiments done on the oncogenic ability of oestrogen in lab animals have resulted in the proliferation of lesions having similar histological features as IM [11]. Research regarding the specific genetic aberration has been limited with monosomy 9q, trisomy 16q, and del(6)(q12;q15) being the few cytogenetic abnormalities reported [12, 13].

The solitary nodules are most commonly seen in the head and neck region which include scalp, forehead, orbit, parotid region, and oral cavity [1, 7]. Although considered the most common tumour of infancy, the reported incidence of solitary osseous myofibromas is rare [14–16]. Apart from the soft tissues and the skeleton, rare involvement of organs like lung, heart, gastrointestinal tract, and pancreas has been reported [17]. The incidence is equal in males and females [2]. Myofibroma manifests as a single swelling or a mass most commonly in the dermis and subcutis which may be freely movable at times. The overlying skin is usually normal, sometimes may resemble a purplish macule and infrequently may ulcerate. In the present case, the swelling was seen as a large nodule measuring 3 × 4 cm, firm, nonmovable mass present over the right frontal bone with a normal overlying skin. 

Radiographically, myofibromas appear as a well-defined unilocular radiolucency in most cases with a few exhibiting multilocularity. Intraosseous lesions may show lytic areas with marginal sclerosis [18]. MRI is useful when dealing with soft tissue tumours especially in children [16]. In the present case, however, T_1_ weighted image revealed a well-delineated osteolytic lesion which appeared hyperintense as compared to the brain which prompted a provisional diagnosis of leiomyoma/eosinophilic granuloma. 

Microscopically, the lesion presented with a biphasic cellular pattern which is usually seen in myofibromas [1, 9, 10]. The tumour cells showed diffuse immunopositivity for SMA (contractile protein actin) and vimentin (a mesenchymal cell intermediate filament) and negative for desmin (Smooth muscle antigen) and S-100 (nervous tissue antigen) which has been consistently used to spot a myofibroblastic lineage [19].

Histopathologically, the definitive diagnosis of a myofibroma was challenging in light of the various differential diagnosis that had to be excluded. Nodular fascitis was excluded as it is rarely seen in infants and has a prominent myxoid matrix and the absence of hemangiopericytoma-like pattern of myofibroma. Leiomyoma and leiomyosarcoma were excluded based on the negativity of tumour cells to desmin. Another closely resembling tumor which was considered was fibrous histiocytoma, which is typically composed of polymorphous cells arranged in a storiform pattern and exhibits only focal staining with SMA. Neurofibroma and neurofibromatosis were excluded based on the immunonegativity of myofibroblasts to S-100. Fibromatosis usually has a monophasic growth pattern consisting of long fascicles of spindle cells among abundant wavy collagen fibres which was not seen in the present case. Fibrosarcoma could be ruled out by the presence of nuclear atypia, high mitotic counts, and abnormal mitosis, which was, however, absent in the present case.

Conservative surgical treatment is the most effective treatment of IM [20]. Some cases show spontaneous regression [21] and thus require no treatment at all. The prognosis is usually excellent with the rate of recurrence being less than 10%. General systemic examination must be undertaken to eliminate the presence of other nodules. The patient should be monitored for at least 5 years to assess recurrences and to exclude the manifestation of further nodules characterizing myofibromatosis. 

## 4. Conclusion

Infantile myofibroma is a benign, self-limiting, and localized tumour consisting predominantly of myofibroblasts. The diagnosis of this apparently benign lesion is important as it may histologically mimic other forms of aggressive fibromatoses. Careful exclusion of IM from these with the help of specific markers may help in accurate diagnosis and appropriate surgical management. The case described here is a very rare type of solitary IM eroding into the frontal bone without intracranial involvement.
